# Abandoned embryos in Brazil: advances in the decisions. Are we there yet?

**DOI:** 10.5935/1518-0557.20180038

**Published:** 2018

**Authors:** Maria do Carmo Borges de Souza, Roberto de Azevedo Antunes, Ana Cristina Allemand Mancebo

**Affiliations:** 1 Fertipraxis Centro de Reprodução Humana - Rio de Janeiro - Brazil

The Brazilian Federal Council of Medicine (CFM) on September 21, 2017, published its new Resolution, number 2168 (CFM 2017), regarding assisted reproduction procedures. Concerning the cryopreservation of gametes or embryos (item V), it defines that cryopreserved embryos can be discarded after three years or more, should it be the expressed will of the patients. In addition, for the first time, it states that this applies to abandoned embryos, as well. They define in a single paragraph that abandoned embryos are those which the owners failed to comply with the pre-established contract, and such persons were not located by the clinic that performed the procedure.

We recognize that this inclusion was not just a simple answer. It resulted from a careful consideration by the The CFM Counselors based on an official consultation sent jointly by the group of clinics from the State of Rio de Janeiro, of which we are part. However, we have additional important goals to discuss and to achieve, looking for excellence in our patient-care best practices (Souza *et al*., 2017).

The First point is that our national data are not clear. The SISEMBRIO/ANVISA (National System of Embryos Production/National Health Surveillance Agency) informs that from 146 reporting centers in 2017, there were 36,307 completed cycles, which produced 78,216 frozen embryos (SISEMBRIO/ANVISA 2017). It also informs that there were 68,891 transferred embryos in the same period. A question remains about the number of embryos cumulatively kept in freezing through the years.

The 26^th^ edition of the RLA/REDLARA (Latin American Registry/Latin America Network on Assisted Reproduction)(Zegers-Hochschild *et al*., 2017), multinational data undertaken in 2014, collected from 159 institutions in 15 countries (54 accredited centers from Brazil) showed 65,534 initiated cycles, with a mean of 2.06 embryos (range 1-6) per transfer, a total of 31,855 fresh and 19,910 frozen embryo transfers. When we perform a thorough analysis on the SISEMBRIO system, we observe the absence of information on the number of both fresh and frozen embryo transfers, which compromises the accuracy of the reports. Such data are already being reported on RLA, which is worldly recognized for its accuracy of information. However, neither system report on abandoned embryos; and to have concrete data on our frozen embryos and the abandoned ones is crucial for a more educated debate in our clinics, in Brazil and in Latin America. So, joining REDLARA should be considered by all Latin American clinics, to strengthen our practices, which does not prevent (even helps) the creation of national registries that will be more robust.

The Second point is related to steps to proceed until the discharge of the abandoned embryos. It demands an effort to contact the owners of these embryos, so they can either have the final decision of discarding or paying for the maintenance storage debts, mainly when there had not been the possibility of such authorization in the old contracts. And, of course, from September 2017 on, all programs should create and enforce written policies on the designation, retention, and disposal of abandoned embryos.

From a third point, The Ethics Committee of ASRM (2013) concluded that a program might dispose of the embryos by removal from storage and thawing without transfer. In no case should embryos deemed abandoned be donated to other couples or be used in research. This kind of statement raises moral issues for which there is no unanimity.

Bob Edwards (Editors and Editorial Staff, 2010) understood and consistently worked on the ethical implications of assisted reproduction techniques. That he left us with a strong legacy. Various recent papers, such as from Fruchter & Shalev (2015), Tonkens (2013; 2016), Pennings *et al*. (2017) have been dealing with these matters.

Interestingly, Tonkens argues that willful embryo abandonment is morally unacceptable, because of the abandoner's unfair treatment of the clinic storing their abandoned embryos, the apparent lack of sympathy for the plight of other people like those responsible parents, who require assistance (e.g. donated embryos) in pursuing their family-building goals and the abandoners' failure to meet their responsibility for directing the handling of their embryo.

The logical implication is that we need to discuss and look for reasonable grounds for justifying a new design of national policies and practices. Perhaps it is time to also reintegrate the discussion on the possibility of allocating them for scientific and/or clinical research before their destruction.
